# Recreational Performance Evaluation of Urban Forests: Spatial, Socio-Cultural, and Public Health-Related Perspectives

**DOI:** 10.3390/ijerph22091401

**Published:** 2025-09-08

**Authors:** Zeynep Pirselimoğlu Batman, Elvan Ender Altay

**Affiliations:** Department of Landscape Architecture, Faculty of Agriculture, Bursa Uludağ University, 16059 Bursa, Turkey; elvanender@uludag.edu.tr

**Keywords:** urban forest, urban ecosystem, public health, recreational performance

## Abstract

Urban forests are natural habitat areas within urban ecosystems that enhance physical, mental, and social well-being. By integrating natural and cultural values into the urban landscape, these areas offer individuals opportunities to interact with nature and engage in various recreational activities. Recreational activities increase physical activity levels, help reduce stress, strengthen mental health, and foster social interaction, thereby significantly protecting and improving public health. This study aims to evaluate the recreational performance of urban forests—an essential component of the urban ecosystem—through a multidimensional approach. In this context, ecological (topography, vegetation, water resources, soil structure, climate), physical (accessibility, infrastructure, area size), social (activity diversity, usage intensity, community events), and cultural (landscape values, urban identity, conservation status of cultural landscapes) factors were considered as key indicators. Bursa Atatürk Urban Forest was selected as the study area, and the methodology integrated SWOT (Strengths, Weaknesses, Opportunities, Threats) analysis with weighted multi-criteria decision-making techniques. In addition, the qualitative data obtained were supported by statistical analysis methods to reveal the relationships among the criteria quantitatively. Through this holistic approach, the recreational performance of the urban forest was evaluated scientifically, leading to the conclusion that the area’s strengths should be preserved, its weaknesses improved, and its cultural landscape values managed sustainably. The study provides a valuable decision-support framework capable of guiding strategic planning for the future.

## 1. Introduction

The key components of urban green infrastructure typology are natural habitats (urban forests, wetlands, etc.), green corridors (such as riparian zones), and open and green spaces (such as parks, residential gardens, etc.). Urban forests, which emerge through the interplay of biotic and abiotic elements forming natural landscape values and the human interactions that shape cultural landscape values, constitute a significant component of the urban green infrastructure system [1]. With the increasing urbanization during the 20th century, trees began to be integrated into urban systems as essential elements of urban settlements. Consequently, a distinct research discipline emerged that could inform and guide the management of all trees within urban areas, which is referred to here as urban green network management [2].

Due to increasing global urbanization and its adverse impacts on urban ecosystems, urban forests have become a focal point for their contributions to sustainability and improving urban life quality [3]. With the rise in urbanization and the consequent loss of natural areas, urban forests have emerged as critical public health components regarding environmental sustainability and their positive effects on individuals’ physical, mental, and social well-being. In this context, urban forests are regarded as nature-based solutions that enhance the quality of life for urban residents, serving as accessible and sustainable health-supporting systems [4,5].

Urban forests situated on the periphery of cities provide direct and indirect benefits to the urban environment and the people who live in it [6]. Based on definitions of urban forestry and urban forests, urban forestry is a specialized discipline that involves planning, designing, establishing, protecting, and managing tree stands and forested areas, either naturally occurring or artificially created within or around cities, for the public good. On the other hand, urban forests are defined as forested areas, either naturally existing or artificially established within or near urban environments, that provide aesthetic and functional value to the urban structure, offer recreational opportunities to city dwellers, and are easily accessible [7]. Additionally, urban forests can be described as forests primarily composed of tall native or exotic tree species, located within or near cities, intended to contribute to the urban climate, ecosystem, aesthetics, and recreation demand [8]. Besides meeting the green space needs of cities and residents, urban forests also serve as recreational areas that support individuals’ connection to nature, promote a sense of belonging to the natural environment, inspire physical/mental well-being and creativity, and offer various leisure opportunities [9].

Urban forests today are recognized for their aesthetic and ecological values—that is, their visual appeal, contribution to landscape beauty, and their role in supporting biodiversity, habitat provision, and ecosystem balance—and as essential components of healthy urban infrastructure. Through their roles in enhancing air quality, mitigating the urban heat island effect, and providing opportunities for physical activity, urban forests contribute positively to urban populations’ overall health and well-being [5,10].

Urban forests are significant multifunctional landscape elements in cities and serve as nature-based solutions to mitigate the adverse socio-ecological effects of urbanization. Their primary function has traditionally been recognized as recreation [11]. Research has demonstrated that recreational activities conducted in natural settings reduce stress levels, enhance cognitive functions, and improve overall life satisfaction. Therefore, the recreational potential of urban forests plays a complementary role in protecting and promoting public health [12,13,14]. Forest areas within or near metropolitan areas have historically been used for various recreational activities. Hence, the primary function of forested areas designated as “urban forests” is recreation [15].

In this regard, recreational planning objectives [7] include:

Scenery (Visual Appeal): The presence of scenic viewpoints and visually appealing landscapes is essential. These features greatly enhance the attractiveness of the site for visitors.

Accessibility: Ease of access is a primary consideration. Urban forest sites should be easily reachable by both private and public transportation.

Recreation: The area should support various recreational activities through its potential size, layout, and suitability for diverse uses, as well as its cultural and aesthetic values, including historical significance, cultural symbolism, visual appeal, and contribution to landscape beauty.

Water Bodies: Areas with water potential are highly preferred. The presence of drinking water sources increases attractiveness, while larger water bodies such as seas, lakes, ponds, and rivers significantly enhance usage potential and visual impact.

Vegetation: Areas rich in coniferous and broadleaf (mixed) tree species should be prioritized for both biodiversity and aesthetic diversity.

The recreational use of urban forests significantly impacts residents’ physical, mental, and social health. These forests provide natural habitats within the urban landscape, allowing people to interact with nature and participate in diverse recreational activities. The concept of ‘cultural landscape values’ encompasses the relational meanings individuals establish with urban forests, including the site’s position in social memory, spatial usage patterns, and place identity shaped by aesthetic perceptions. In addition, specific physical attributes—such as transportation infrastructure and recreational facilities—should also be regarded as spatial representatives of this interaction network and thus considered among the components of cultural landscapes.

The importance of recreational use in urban forests can be summarized as follows: Urban forests offer ideal spaces for outdoor activities such as walking, jogging, and cycling. Spending time in nature helps reduce stress and alleviates mental health issues such as depression and anxiety. These forests provide social spaces for families, friends, and communities to gather and spend time together. They also contribute to preserving city biodiversity, raising environmental awareness, and offering educational opportunities related to nature. Moreover, urban forests improve air quality, increase carbon sequestration, reduce noise pollution, and help regulate urban temperatures, thus maintaining ecological balance and enhancing quality of life. They offer recreational options for individuals seeking an escape from the monotony of urban life, supporting various activities such as picnicking, nature walks, and birdwatching. In conclusion, the recreational use of urban forests is critical in promoting healthy, balanced, and fulfilling lives, fostering deeper connections between people and nature, and supporting the conservation of urban ecosystems.

As cities continue to grow, green spaces gain even more critical importance. In this context, the sustainable management of urban forests—particularly from a recreational use perspective—has become an increasingly vital research area. Numerous researchers, including Gül et al. [16], Fuller et al. [17], Uslu & Ayaşlıgil [7], Yılmaz et al. [18], Beatley [19], Kurdoğlu & Düzgüneş [15], Wolch et al. [20], Eminağaoğlu et al. [21], Uzun & Ayhan [22], Chen & Li [11], Yeşil & Güzel [23], Uygur & Özkan [24], Akyıldız [9], Pişkin & Seyidoğlu Akdeniz [25], Seyhan & Bulut [26], Ertem Mutlu & Cengiz [27], and Ender Altay & Pirselimoğlu Batman [28], have conducted studies focusing on the recreation and recreational potential of urban forests. These studies generally emphasize the positive impacts of urban forests on quality of life and highlight the necessity for more effective planning and management approaches. There is a research gap regarding the effects of human activities on biodiversity and ecosystem functions, particularly about how different recreational activities—those associated with natural landscape values (geological and geomorphological structure, topographic features, natural vegetation, hydrology, climatic characteristics, soil structure, and geographical location) and cultural landscape values (current land use, accessibility and road networks, visual qualities, other environmental factors, boundaries and security, and recreational facilities)—can contribute to the development of sustainable use strategies, as well as how the performance of these activities within the area can be enhanced. In addition, studies directly assessing cultural values reveal significant conceptual divergences. It is particularly emphasized that cultural landscape components should be addressed in their spatial, perceptual, and historical dimensions, and that these values encompass not only tangible cultural heritage but also the emotional and functional bonds that individuals establish with the area. The recreational performance of urban forests, when evaluated based on natural and cultural landscape values through an integrated assessment of ecological, physical, social, and cultural factors, provides a comprehensive framework for both the sustainable use potential of the area and user satisfaction.

Although the existing literature provides significant insights into urban forests’ ecological, physical, social, and cultural values, comprehensive studies that assess their recreational performance within an integrated public health framework using multidimensional criteria remain limited. In particular, there is a notable gap in research examining the relationship between different types of recreational activities and the natural and cultural landscape character, especially regarding their impacts on biodiversity and ecosystem functions. Furthermore, integrating natural and cultural landscape data with public health indicators into decision-support models is scarce. The simultaneous intensification of urbanization pressure, loss of natural areas, and city public health challenges underscores the scientific and practical urgency of addressing these gaps. This study aims to respond to this need by offering an innovative and applicable contribution to both academic literature and urban planning and health policy fields, while supporting strategies that promote sustainable use and enhance the performance of recreational activities.

In this context, this study aims to assess the recreational performance of Bursa Atatürk Urban Forest through a multidimensional approach, analyzing the ecological, physical, social, and cultural factors that influence this performance, and developing strategic management recommendations based on the results. Accordingly, the study integrates SWOT analysis with weighted multi-criteria decision-making techniques, supporting qualitative data with quantitative methods to construct a strategic evaluation model. Integrating SWOT analysis with numerical methods allows for more objective and measurable outcomes by reducing subjectivity. Moreover, SWOT scores were further analyzed using *t*-tests, ANOVA, and correlation analysis to explore interrelationships among criteria. This comprehensive approach enables the scientific evaluation of urban forest recreational capacity and supports the spatial planning of natural and cultural landscape data. In this study, cultural landscape values were defined within the context of planning and recreation, encompassing parameters such as accessibility, land use, recreational facilities, safety, and visual aesthetics, thereby representing the socio-cultural dimension of the urban forest. Consistent with previous research emphasizing the integrated nature of cultural landscapes, the physical indicators addressed under this category were considered not merely technical attributes but cultural elements with social representativeness. The results obtained provide valuable input not only for site management but also for environmental and health policies that support urban residents’ right to a healthy life. In this regard, the study offers an integrated urban planning perspective within the context of public health, based on nature-based solutions. Moreover, this study aims to contribute to conceptualizing cultural landscape values by arguing that such values can be redefined not only in historical and aesthetic terms but also based on social accessibility and spatial functionality.

## 2. Materials and Methods

### 2.1. Material

**Figure 1:**
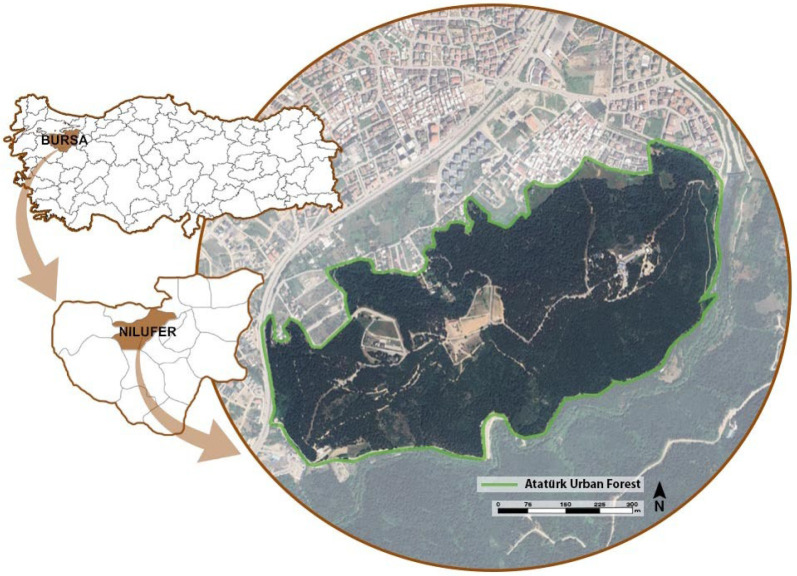
Location of study area [30].

The primary material of this study is the Bursa Atatürk Urban Forest, located in the Nilüfer district of Bursa province. In addition, the literature related to the area, maps, plans, and visual data obtained through fieldwork constitute the other materials used in the study. Bursa Atatürk Urban Forest, with its area of 150 hectares, is one of the significant open green spaces that meets a large portion of Bursa’s demand for green areas. The results obtained provide valuable input not only for site management but also for environmental and health policies that support urban residents’ right to a healthy life. In this respect, the study offers an integrated urban planning perspective within the context of public health, based on nature-based solutions. Accordingly, the sustainable conservation and effective management of Atatürk Urban Forest are of great importance for both the continuity of ecosystem services and the enhancement of the quality of life of city inhabitants. (Figure 1). Situated approximately 12 km from the city center, the average elevation of the forest is 205 m. Atatürk Urban Forest offers substantial value for conserving natural habitats and recreational use [28]. Atatürk Urban Forest is located in the Nilüfer district of Bursa. According to 2023 data from the Turkish Statistical Institute (TÜİK), the population of Nilüfer district is 543,934 [29].

### 2.2. Method

This study examined the Bursa Atatürk Urban Forest through qualitative and quantitative approaches. The research method was developed by integrating various analytical techniques. The method integrates ecological and socioeconomic factors within the recreational area and landscape planning framework. In this process, on-site observation, the literature review, and the weighted criteria were employed [31,32,33,34]. To evaluate the recreational performance of an urban forest, a weighted multi-criteria evaluation method integrated with SWOT (Strengths, Weaknesses, Opportunities, and Threats) analysis was utilized. This method combines qualitative and quantitative data for strategic analysis and decision support.

**Figure 2:**
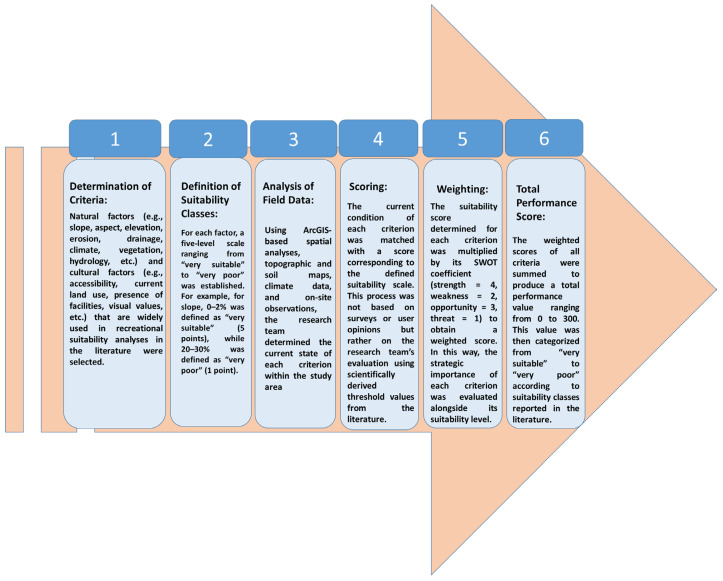
Flow chart.

This method eliminates variations based on subjective user opinions, delivering repeatable, comparable, and scientifically grounded results. One of the study’s strengths is that it presents a standardized assessment model applicable to different areas. This highlights the significance of the study’s methodology (Figure 2).

Initially, a literature review was conducted to identify urban forests’ natural features, including geological and geomorphological structure, geographical location, topographical features, natural vegetation cover and wildlife, hydrology, climatic conditions, soil structure, and cultural landscape values, including accessibility, current land use, other environmental characteristics, boundaries- security and recreational facilities. The area’s current status was determined through field surveys, direct observation, interviews, and literature analysis. Using the existing site data, the recreational potential value of the area—defined as the overall capacity to support diverse recreational activities through the integration of ecological, social, physical, and cultural factors within the framework of its natural and cultural landscape values—was determined. This definition also encompasses the extent to which the area contributes to public health through factors such as opportunities for physical activity, stress reduction, psychological restoration, and social interaction. This assessment reveals the extent to which the area functions effectively regarding factors that directly impact public health, such as physical activity, stress reduction, and interaction with nature. Using the existing site data, the recreational potential value of the area was assessed, and suitability levels were calculated using the weighted criteria method. The spatial data of the study area were used to measure its recreational potential.

In this stage, evaluation factors and sub-factors suitable for recreation were determined [16,21,35,36,37,38,39,40,41,42,43,44,45] and scored using existing site data.

The Criteria influencing recreational performance were determined based on factors analyzed under natural landscape values and cultural landscape values, including slope, aspect, elevation, erosion, drainage, land capability classification, climate, hydrology, vegetation cover, area size, current land use, accessibility, road type, pedestrian paths, bicycle paths, facilities for people with disabilities, availability of parking areas, other environmental factors, security, and recreational facilities. These criteria were intentionally aligned with determinants of public health to emphasize the link between environmental quality and human well-being.

The evaluation factors determined according to recreational suitability potential were scored on a scale of 1 to 5: 5 = Highly Suitable, 4 = Suitable, 3 = Moderate, 2 = Low, 1 = Very Low. These scores were derived from relevant sources in line with recreational suitability measurements [16,21,40,41,42]. The scoring was performed based on expert opinions and field data, considering the existing characteristics of the area.

In the next stage, various criteria such as the natural and cultural landscape values (including geological and geomorphological structure, geographical location, topographical characteristics, natural vegetation cover and wildlife, hydrology, climatic characteristics, soil structure, accessibility, current land use, and recreational facilities) were defined. The natural and cultural values were analyzed to identify the factors and sub-factors. Each criterion and subcategory was evaluated and classified according to the SWOT analysis. A suitability score (between 0 and 4) was assigned to each sub-factor under the relevant SWOT category based on its level of appropriateness.

In this process, the scoring was not based on surveys. Still, it was carried out by the research team’s field observations, spatial analyses, and threshold values obtained from the literature. This way, the assessment was freed from variations arising from subjective user statements, thereby gaining a repeatable, comparable, and scientifically grounded structure.

Each criterion was evaluated based on SWOT data and scored accordingly. Each criterion’s average score was calculated to obtain a single representative value.

- Strengths (S): Internal positive factors that provide advantages to the area (Positive Value)—Score: 4
- Weaknesses (W): Internal negative factors that represent limitations or deficiencies (Negative Value)—Score: 2
- Opportunities (O): External positive factors indicating potential future development (Positive Value)—Score: 3
- Threats (T): External negative factors representing risks or dangers (Negative Value)—Score: 1

The weighted score for each sub-factor was calculated by multiplying its suitability score by the average SWOT coefficient:

Weighted Score = Suitability Score × Average SWOT Coefficient

Based on the suitability values, the total recreational performance score of the area was evaluated according to the following classification: 300–240: Highly Suitable, 239–179: Suitable, 178–118: Moderate, 117–57: Low, 56–0: Very Low.

In this study, the weighted evaluations employed in the SWOT and MCDM (Multi-Criteria Decision Making) methods were not based solely on a single perspective; instead, they were determined using scales that have been applied in different geographical and social contexts and are recognized in the literature for their reliability [16,21,40,42,45]. Each criterion and sub-criterion was scored using the standard definitions and suitability classes provided in these studies. Consequently, the evaluation process was grounded in field observations and objective references from multiple research sources. This multi-source approach reduced the risk of subjectivity and provided a methodologically replicable framework. Furthermore, the explicit presentation of the criteria and scoring ranges adopted from the literature ensures that the same method can be reapplied under similar conditions in different regions.

The results obtained from the evaluations were statistically analyzed using SPSS version 28. To determine whether the differences between the weighted scores of natural and cultural data were statistically significant, an independent samples *t*-test—a parametric test used to compare the means of two independent groups—was applied [46]. This test assesses whether the differences in group means are likely due to chance.

Additionally, a one-way analysis of variance (ANOVA) was used to identify significant differences among individual parameters within natural and cultural datasets. One-way ANOVA is a method used to test whether there are statistically significant differences between the means of three or more independent groups [47]. Following the ANOVA results, the Duncan multiple comparison test was applied to determine the specific group differences. The mean values obtained from the Duncan test were ranked from highest to lowest and categorized using letters, with statistically significant differences reported at *p* ≤ 0.05 and *p* ≤ 0.001.

Furthermore, a correlation analysis was conducted to determine the relationships among the variables derived from the SWOT analysis scores. Correlation analysis is a statistical method used to identify whether a meaningful relationship exists between two or more variables and to reveal the relationship’s direction (positive or negative) and strength (degree). As a result of the analysis, the levels of association between variables were assessed, and statistical significance was indicated at *p* ≤ 0.05 and *p* ≤ 0.001 levels [48].

By integrating these diverse analytical methods, the study not only quantified the recreational capacity of the site but also established a conceptual framework linking environmental quality, accessibility, and cultural amenities with public health benefits.

As a result of all analyses, the factors that enhance performance in the area and those requiring improvement have been identified. Given that interventions aimed at strengthening recreational potential are reported to benefit individuals’ physical and mental health, such enhancements are theoretically anticipated to contribute to public health. Nevertheless, public health outcomes are not directly measured in this study; therefore, this anticipated contribution is not empirically examined within the scope of the present methodology.

Based on the results of all analyses, the specific factors contributing to enhanced performance and those requiring improvement within the area were identified. This study’s systematic and comparable findings were evaluated as valuable inputs for site management, nature conservation, spatial planning, and recreational design processes. Accordingly, these analyses provide a solid scientific foundation for decision-makers and practitioners in developing site-specific strategies and planning sustainability-oriented interventions.

## 3. Results

This study aims to evaluate the urban forest’s recreational performance in a multidimensional manner based on ecological, physical, social, and cultural factors. These four thematic categories have been structured to encompass the components of natural systems and user experience, each translated into measurable and interpretable criteria. The analyses presented in the findings section have been structured to encompass these four themes. Ecological factors were assessed through natural landscape values (geological and geomorphological structure, topographic structure, vegetation cover, and wildlife, hydrology, climatic characteristics, and soil) by analyzing related factors and sub-factors such as slope, aspect, elevation, erosion, drainage, land capability class, climate, hydrology, and vegetation cover. Similarly, cultural landscape values were assessed by analyzing data on existing land uses, accessibility, visual values, and recreational amenities, which were then evaluated under cultural, social, and physical factors. In this study, cultural factors were not confined solely to historical or artistic elements but were broadened to encompass the experiential, functional, and symbolic relationships individuals establish with natural spaces. Accordingly, elements such as accessibility, land-use patterns, visual attractiveness, and recreational infrastructure were evaluated as indicators reflecting the cultural landscape values of the site and as features that generate cultural meaning for users. These elements become integral to the cultural factor through the meanings attributed to the space, use patterns, a sense of social belonging, and the symbolic bonds formed with the environment. Within conceptualizing and operationalizing cultural factors, measurable indicators such as accessibility, land use categories, recreational infrastructure, safety conditions, and visual quality were employed. These criteria were integrated with ecological, physical, and social factors, thereby establishing a comprehensive analytical framework for the multidimensional assessment of the recreational performance of the urban forest.

These data were spatially analyzed, mapped, and associated with suitability scores. Physical factors were examined regarding the site’s accessibility, existing infrastructure (e.g., roads, parking facilities), and other functional characteristics. Social factors were considered, including the potential diversity of user activities, recreational amenities, and safety indicators. Cultural factors were evaluated together with visual aesthetics and viewpoints. Through this approach, the assessment of the urban forest’s recreational performance based on natural and cultural landscape values revealed how ecological data reflect the site’s natural capacity, physical data represent accessibility and infrastructure adequacy, social data indicate diversity of use, and cultural data shape the aesthetic dimension of the area.

### 3.1. Natural and Cultural Resource Values of the Study Area

This section identified the area’s existing natural and cultural landscape values to measure its recreational performance. These data were subsequently evaluated using the determined factors and sub-factors.

**Geographic Location and Area:** Atatürk Urban Forest is located between latitudes 40°11′14.0″ N and longitudes 28°59′05.3″ E, covering an area of 150 ha. The forest lies on the northwestern rim of Uludağ, adjacent to the city’s periphery, bounded by the Gümüştepe district and the village of Misi. The Nilüfer River forms part of its boundary, while the nearby Hüdavendigar Park further contributes to its significance as a green buffer between Bursa and Uludağ [30].

**Geology and Geomorphology:** The site comprises the northern-facing slopes of Uludağ, underlain by a low-angle Bursa fault—a typical triangular fault-facet geometry. Deeply dissected by northward-flowing parallel streams, the flanks reach elevations up to 1000 m. Eocene and older rock units, along with Miocene lacustrine-fluvial deposits, outcrop in the northern slopes, creating steep relief on older formations and flatter topography on younger ones [49,50,51].

**Topography:** The steepest slopes occur in the southern section, while the terrain flattens inward. Approximately 61.99 ha (53%) of the area has 12–20% slopes, followed by 27.66 ha (23%) with 20–30% slopes. Predominant aspects are south, southwest, north, and northwest, with southeast-facing aspects covering 20%. Elevations mostly range between 200 and 250 m (36%) as per 1:25,000 topographic maps.

**Figure 3:**
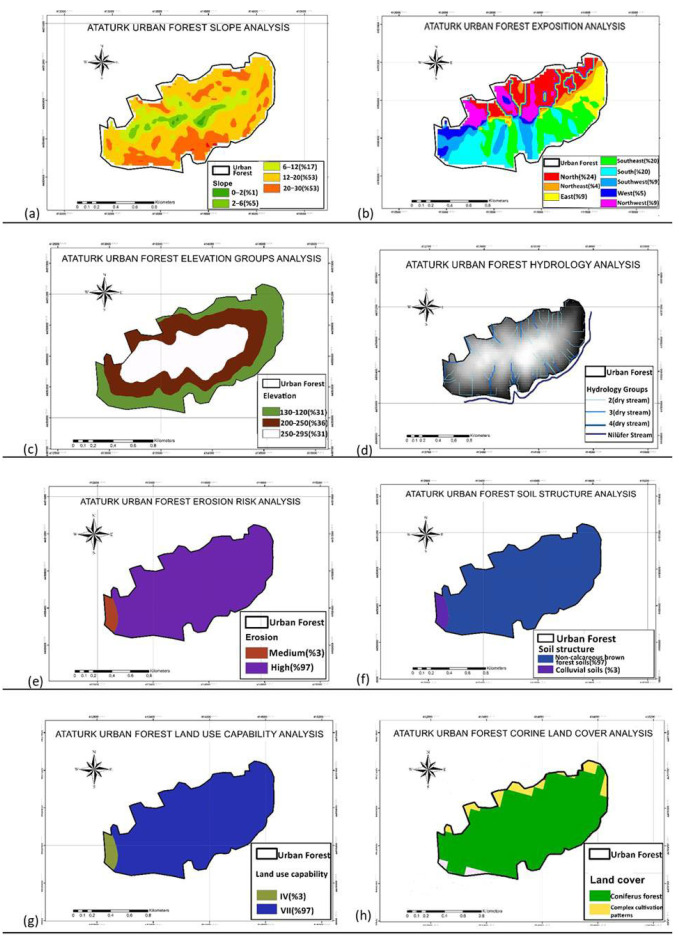
(**a**) Slope analysis; (**b**) Exposition analysis; (**c**) Elevation groups analysis; (**d**) Hydrology analysis; (**e**) Erosion risk analysis; (**f**) Soil structure analysis; (**g**) Land use capability; (**h**) Current land cover status.

**Flora and fauna:** The forest is among Marmara’s most biodiverse areas, dominated by Turkish Pine (*Pinus brutia*), with significant stands of Black Pine (*P. nigra*), Oak species (*Quercus robur*, *Q. petraea*), Oriental Hornbeam (*Carpinus orientalis*), and European Chestnut (*Castanea sativa*). Scots Pine and Spruce are in cooler pockets, alongside *Robinia pseudoacacia*, *Fraxinus* spp., and evergreen shrubs like laurel and *Arbutus*. Understory vegetation includes heather, thyme, sage, ivy, and other species, supporting a rich biotic community [52] (Figure 3).

**Hydrology:** The Nilüfer River flows along the southern and eastern borders, with additional ephemeral streams traversing the interior.

**Climate:** The region enjoys a mild Marmara climate. Bursa’s annual mean temperature is 12.6 °C, with August peaking at 22.5 °C and January lows averaging 2.6 °C. The relative humidity averages 58%, and annual precipitation is approximately 893 mm, highest in winter and spring, lowest in early summer. Prevailing winds include northeasterly (poyraz), southwesterly (lodos), and northwesterly (yıldız), with February being the windiest month (avg. 13.4 km/h) [53,54].

**Soil Characteristics:** Soils are predominantly non-calcareous brown forest and colluvial types. About 97% of the area exhibits severe erosion and is classified as Agricultural Capability Class VII (very steep, erosion-prone soils usable only under strict management [55] (Figure 2).

**Accessibility:** Situated 12 km from Bursa city center within the Nilüfer district, the forest can be accessed partially by public transport and easily by private vehicle. Walking and vehicle paths are in place, with separate entry/exit points, security presence, and parking lots. However, accessible design for disabled users and dedicated bicycle paths are lacking.

**Land Use:** The area is exclusively forested; no residential units are inside, though neighboring settlements include Odunluk and Gümüştepe.

**Visual Landscape:** Thanks to its varied topography and dense forest cover, the site offers multiple panoramic vistas, resulting in high landscape value.

**Other Environmental Factors:** No in situ air, water, or noise pollution was detected. However, nearby urban structures and transit roads pollute the ambient air and noise. Certain sections along the Nilüfer River exhibit signs of pollution.

**Safety:** A controlled access system is in place, but there are no physical barriers delineating the boundary of the forest within the urban system.

**Recreational Facilities:** Bursa Atatürk Urban Forest is located in the Odunluk and Gümüştepe neighborhoods of Nilüfer District, Bursa, covering a total area of 150 hectares. The Nilüfer River runs along the southern and eastern borders of the forest, forming a natural boundary. Situated in the eastern part of Nilüfer, Atatürk Urban Forest is an important recreational area for those seeking to engage with nature. The forest area is one of the region’s prominent natural open spaces, offering extensive walking trails, picnic areas, playgrounds, and various outdoor activities. The urban forest is located near the northern slopes of Uludağ and provides multiple opportunities to individuals seeking to escape city noise and spend time in nature. In this capacity, it functions as an essential natural therapeutic environment contributing to individuals’ mental and physical health by alleviating stress [28].

Additionally, a privately operated facility within the forest hosts adventure- and nature-based activities. From a structural landscape perspective, the forest includes vehicular roads, walking paths, sidewalks, ramps, and parking areas. However, there is no designated bicycle lane. The ground surfaces of these circulation routes are composed of interlocking paving stones of various colors and asphalt. Other landscape elements within the forest include seating units, lighting fixtures, shading structures, trash bins, fire safety boxes, boundary elements, kiosks, a bicycle station, fountains, and children’s play areas with associated equipment. Observational data indicate that user density increases significantly during the summer, while the site experiences low visitation during winter due to weather conditions. Moreover, the area is more heavily used on weekends than on weekdays during the summer. During the school year, educational institutions organize field trips to the forest, and individuals of different age groups engage in various recreational activities.

Natural and cultural data were collected, processed, and used to produce thematic maps. A 1:25,000-scale soil map of Bursa Province was digitized using ArcGIS 10.8 software to create maps indicating land capability classes, soil types, and erosion risk for the study area. Similarly, 1:25,000-scale topographic maps were digitized to generate elevation, slope, and aspect maps. CORINE 2018 land use data were used to identify the current land use status of the study area (Figure 3).

### 3.2. Evaluation of the Recreational Performance of the Urban Forest

The recreational performance of the urban forest was evaluated based on the recreational suitability potential using the Weighted Criteria Method. This assessment was conducted by drawing on the studies of Gül et al. [16], Akten [39], Akten et al. [40], Mansuroğlu and Baytekin [41], Cengiz and Gönüz [42], Uzun et al. [43], Eminağaoğlu et al. [21], and Gökyer and Tekiner [44]. This evaluation also utilized maps from the natural and cultural landscape data to support the analysis (Table 1).

**Figure 4:**
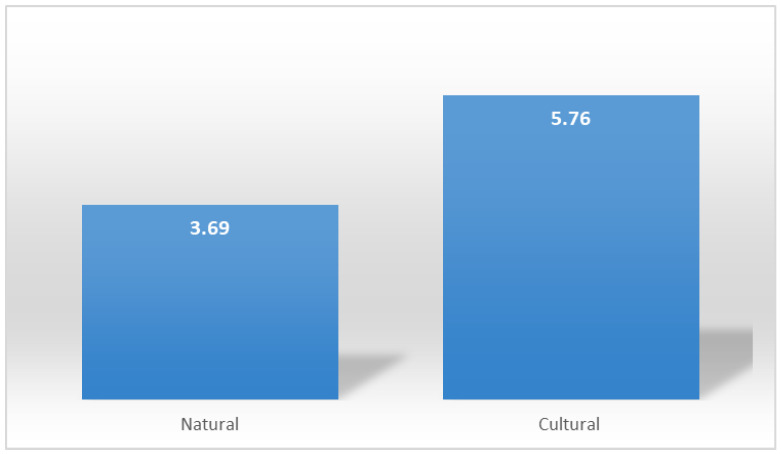
Scores of natural and cultural parameters.

According to the weighted SWOT analysis, the urban forest’s total recreational performance score was 113.5. This score was derived from the study of 24 different factors. The maximum performance score that can be obtained through the evaluation of these 24 factors is 300. Accordingly, the recreational performance of the area was determined to be at a moderate level. Within the methodological framework of this study, the assessment of recreational performance was primarily based on the classification of natural and cultural landscape values, each examined through various subcategories. This classification was adopted to systematically present the area’s ecological, physical, and socio–cultural dimensions. Figure 3 was prepared to more clearly illustrate the influence of these main factors on recreational performance. The Weighted scores of natural and cultural data were compared using an independent samples *t*-test and found statistically significant at the *p* ≤ 0.001 level. Based on the results, the mean scores for cultural factors were higher than those for natural factors. This suggests that cultural attributes, such as land use, visual aesthetics, social functionality, and accessibility, might influence user perception and satisfaction regarding the site, which could be further investigated in future research (Figure 4).

**Figure 5:**
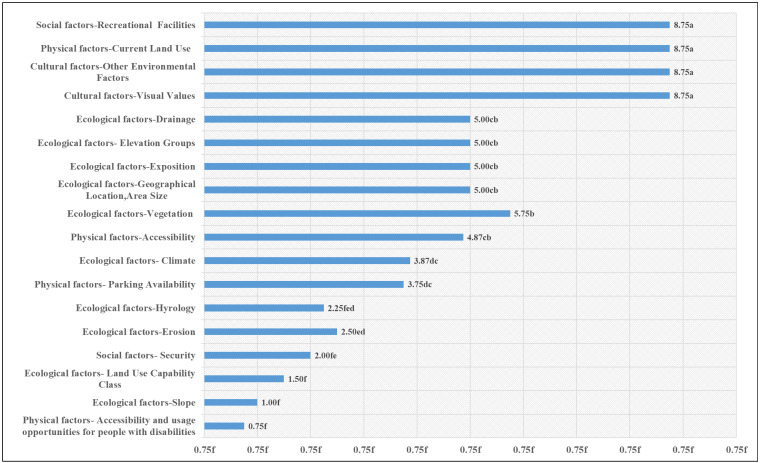
One-Way ANOVA results between natural and cultural parameters. Letters indicate significantly different groups at *p* ≤ 0.05 and *p* ≤ 0.001 levels.

When natural and cultural parameters were jointly compared using one-way analysis of variance (ANOVA), the results were statistically significant at the *p* ≤ 0.001 level. The highest scores were associated with current land use, recreational facilities, visual values, and other environmental factors, each receiving a score of 8.75. This indicates that the functional and aesthetic qualities of the area are at a high level, and these features appear to be associated with the perception and preference of the space, suggesting a potential direction for future research. On the other hand, ecological factors involving natural landscape values such as vegetation cover, area size, aspect, drainage, and elevation groups scored in the 5.00–5.75 range. This suggests that while natural environmental characteristics are essential, the scoring outcomes indicate that functional and visual elements were significantly more critical within the evaluation framework. The lowest score, 0.75, was assigned to the area’s accessibility for individuals with disabilities, indicating that it falls short in meeting their needs and expectations. Ensuring accessibility for all users with physical disabilities is fundamental in creating sustainable and socially equitable public spaces (Figure 5).

**Figure 6:**
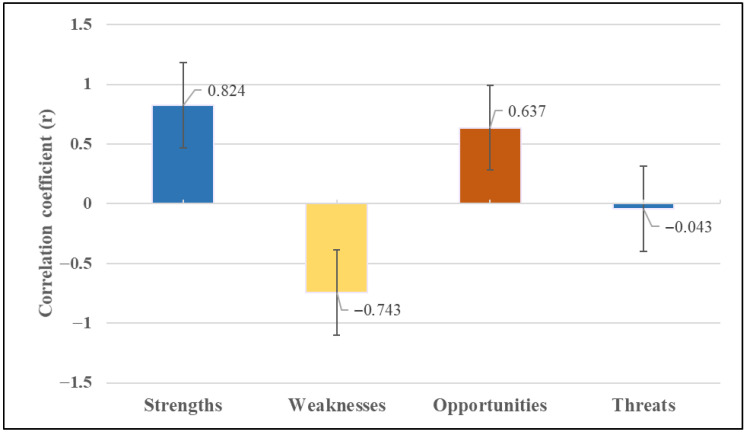
Correlation between score values and SWOT parameters.

The SWOT analysis data of the area were compared using correlation analysis, and it was found that the score values were positively correlated with strengths and opportunities. At the same time, they showed a negative correlation with weaknesses (Figure 6). Accordingly, although strengths and opportunities are positive factors that increase the total score, the negative correlation with weaknesses indicates that increasing weaknesses significantly reduces the total performance score. Furthermore, a negative correlation was observed between strengths and weaknesses, suggesting that rising weaknesses lead to decreased strengths. Eliminating the shortcomings in the area would enhance the effectiveness of the strengths and improve the overall evaluation score. No statistically significant relationship was found between threats and the other parameters (Table 2).

This study’s biophysical, geomorphological, climatic, and infrastructural conditions were not merely described; these data were directly integrated into the recreational performance assessment process through SWOT analysis and multi-criteria decision-making (MCDM) techniques. Each environmental parameter was evaluated within the scope of recreational suitability scoring, thereby quantitatively revealing the influence of physical and ecological characteristics on user experience dimensions such as social use, accessibility, aesthetic value, and comfort. Furthermore, *t*-tests, ANOVA, and correlation analyses were employed to examine the relationships between natural and cultural data statistically, identifying functional links between environmental variables and factors that support public health (e.g., accessibility, visual quality, opportunities for physical activity). In this way, the role of ecological conditions was demonstrated scientifically in terms of spatial planning, and they might also contribute to enhancing social well-being and public health, which could be further explored in future research.

## 4. Discussion

The methodological framework employed in this study integrates SWOT analysis, weighted multi-criteria decision-making (MCDM) techniques, and statistical analyses to holistically evaluate the natural and cultural landscape character and the diversity of recreational activities. While quantitative methods objectively reveal the area’s physical, ecological, and infrastructural capacity, qualitative observations and literature-supported evaluations contribute to a deeper understanding of user experience and perception. In this way, the limitations of an approach based solely on numerical scoring have been reduced, and some of the impacts on public health have been interpreted through both spatial data and socio-cultural context. This integration has enabled the multidimensional and balanced analysis of complex relationships, allowing user-oriented evaluations to be reflected in spatial planning and management decisions. At this point, integrating cultural landscape values into the model has provided a significant advantage in understanding the user–place relationship and planning recreational needs in a multidimensional manner. Beyond the functional use of the space, cultural factors have also enabled the inclusion of users’ sense of belonging, identity, and experiential connections with the area in the analysis.

With the rapid increase in urbanization, the demand for green spaces has risen correspondingly. In this context, the sustainable management and scientific assessment of the recreational capacities of urban forests have gained importance for both environmental sustainability and the protection of public health. In this study, through analyses focused on the Bursa Atatürk Urban Forest, recreational performance was examined from multiple perspectives—evaluating the ecological, physical, social, and cultural factors influencing it. While this study aligns with existing literature [7,15,21,42], it uniquely contributes by integrating SWOT analysis with multi-criteria decision-making techniques.

The model employed in the study provided a detailed assessment of natural factors—such as topography, vegetation, water resources, climate, and soil—as the primary determinants of recreational performance. Topographic features (slope, aspect, elevation) shape the accessibility, safety, and scenic experience of outdoor hiking and cycling. Vegetation enhances thermal comfort by providing shade and influencing the microclimate, while its rich species diversity enables special-interest recreation such as nature observation and photography. Water resources (Nilüfer Stream and the seasonal dry creek) function as visual attractions and focal points for picnic and rest areas, thereby increasing the site’s appeal. Climatic conditions determine seasonal usage intensity, with temperature, sunshine duration, and precipitation patterns making shaded areas and water features particularly critical. Soil characteristics and land use capabilities define which zones can be opened to intensive use and inform management strategies for mitigating erosion risk. These factors were incorporated into the multi-criteria decision-making model through a weighting process, thereby quantitatively revealing the influence of ecological and physical conditions on recreational potential. Nevertheless, the impact of these physical environmental conditions on users can be more accurately evaluated when considered not solely based on natural data, but also in conjunction with cultural variables such as spatial perception, visual quality, symbolic meanings, and social experiences. Accordingly, incorporating cultural factors indicates that the model embodies a planning approach that is both nature-based and human-centered. It is structured around environmental, physical, social, and cultural themes, each represented by specific and measurable indicators. For example, ecological factors such as slope, aspect, vegetation cover, and water presence influence the site’s natural capacity, while physical factors such as accessibility and infrastructure determine its usability. Social factors such as diversity of activities and safety shape user experience and inclusiveness, whereas cultural factors such as visual aesthetics and cultural heritage contribute to the identity and attractiveness of the area. Moreover, these factors are shaped by the meanings, memories, and habits that individuals attribute to space; thus, the cultural landscape is regarded not merely as a legacy inherited from the past, but also as a dynamic construct that users actively reproduce. This condition demonstrates that urban forests function as areas of nature conservation and spatial representations of urban identity and social cohesion. This thematic distinction allows a more integrated interpretation of recreational performance about the natural and cultural landscape values. In this study, ‘cultural landscape values’ do not merely denote heritage or artistic elements; rather, they have been operationalized as landscape-based parameters reflecting human use, perception, and safety. In this respect, they complement the ecological, physical, cultural, and social dimensions.

By integrating SWOT analysis with Analytical Hierarchy Process (AHP) and other quantitative methods, the data became more objective and measurable, thus reinforcing the scientific basis for strategic decision-making. Studies such as Akten et al. [40] and Li et al. [56] emphasize the importance of analytical methods in evaluating recreational spaces. This research demonstrates the practical feasibility of applying these methodologically robust approaches in the context of urban forests.

In-depth statistical analysis of SWOT scores—using *t*-tests, ANOVA, and correlation analyses—unveiled the interrelationships among criteria, enabling a scientific evaluation of the site’s strengths and weaknesses and their mutual interactions. This multi-dimensional assessment aligns with the participatory, voting-based mixed methods proposed by Pomatto et al. [57], further validating the robustness of the evaluation model.

Bursa Atatürk Urban Forest’s natural and cultural landscape values—such as high biodiversity, proximity to a reservoir, and easy accessibility—were identified as core strengths, offering significant recreation opportunities. In particular, cultural landscape values are shaped not only by the physical attributes of the area but also by the social practices, rituals, and habits of its users; hence, safeguarding these assets constitutes an integral dimension of sustainable planning frameworks. However, the infrastructure was found inadequate relative to high summer visitation, reflecting a demand–supply imbalance similar to the case identified by Yılmaz et al. [18] in Artvin. Accordingly, this study recommends strategically reassessing capacity and usage patterns to manage peak-season demand effectively.

The presence of infrastructure deficiencies, such as the lack of bicycle paths and accessibility for individuals with disabilities, limits user diversity and reduces inclusivity. This situation particularly hinders equitable access to urban forests across all age and ability groups, thereby restricting opportunities for physical activity and social interaction that support public health. Such a condition also constitutes a barrier to cultural participation, as the limited physical accessibility of the space restricts the capacity of different social groups to establish cultural ties with the area and to articulate their identities. In this sense, accessibility should be regarded not only as a physical concern but also as an issue of cultural equity. Likewise, it reduces inclusivity, echoing Akten et al. [40] and Özer & Pirselimoğlu Batman [58], who emphasize considering different user requirements during site planning. The forest’s protected status also requires a sensitive management approach that balances conservation and recreation. Ecological planning principles proposed by Cengiz & Gönüz [42] for Çanakkale offer an applicable framework for this site.

Another distinctive aspect of this study is the development of strategic recommendations based on qualitative and quantitative findings, supported by numerical data within a stakeholder-based evaluation process. Similar to the approaches of Pirselimoğlu and Demirel [59] and Gkoltsiou and Paraskevopoulou [60], the opinions of various stakeholders were integrated using SWOT/TOWS (Threats, Opportunities, Weaknesses, Strengths) matrices to propose managerial strategies for recreational and touristic use. Thus, the study assesses the current situation and offers forward-looking spatial planning proposals. The developed strategies aim to enhance the urban forest’s functions that support public health and to ensure that a broader segment of the population can benefit from these areas in a balanced and healthy manner.

Within the scope of this study, no direct user survey or behavioral observation data were collected. The primary aim of the research is to assess the recreational performance of the Bursa Atatürk Urban Forest through a multi-criteria evaluation model based on ecological, physical, social, and cultural factors. Findings related to user satisfaction and seasonal variations in use are not derived from direct survey data; instead, they are grounded in spatial–functional analyses based on field observations and evaluation criteria whose reliability has been well established in the literature [16,21,39]. This approach minimizes the influence of temporary conditions (such as seasonal changes or momentary experiences), providing a replicable and comparable assessment framework independent of contextual factors. Consequently, the results obtained are not bound to a specific period or short-term user experiences, but are suitable for long-term application in planning and management processes.

Nevertheless, the methodology employed in this study presents certain limitations. First, the SWOT- and MCDM-based weightings were derived from field observations and criteria obtained from the literature; as these processes relied on expert evaluations, they inherently carry a certain degree of subjective judgment risk. The absence of direct primary data collection regarding user satisfaction and experiences (e.g., surveys, focus group interviews) limits the analysis’s inclusiveness regarding social perceptions and preferences. This limitation becomes particularly evident in the more in-depth analysis of cultural factors. Qualitative cultural data, such as the meanings users attribute to the space, past experiences, and symbolic connections, can be considered a significant research gap for future studies. Furthermore, applying the model in different geographical, ecological, and socio-cultural contexts may require adaptation to local conditions. In particular, the weighting of criteria and the scoring scales should be adjusted by considering each site’s unique environmental and social dynamics. While these limitations do not preclude the method’s adaptability to different urban forests, they necessitate context-sensitive revisions in each new application.

Given that urban forests are ecological reserves and cultural landscapes reflecting urban identity, planning efforts in these areas must be conducted through a multi-layered and socio-culturally informed framework. As the Bursa Atatürk Urban Forest exemplifies, visual aesthetics, collective memory, usage patterns, and symbolic values shape users’ attitudes and expectations toward the space. Accordingly, incorporating cultural factors into planning necessitates an approach that addresses physical needs, spatial identity, social cohesion, and the long-term sustainability of cultural values.

In conclusion, this study’s holistic and analytical methods present a novel approach model for evaluating urban forests’ sustainability and recreational performance. Compared to similar studies, this model offers a unique and practical framework regarding methodological diversity and strategic planning recommendations. The adaptability and applicability of this approach to different urban forests provide an essential reference for future research. Additionally, by considering the positive effects of urban forests on public health, this approach emphasizes the role of nature-based solutions in enhancing urban quality of life.

## 5. Conclusions

In this study, the evaluation of recreational performance was conducted within a multidimensional framework comprising ecological, physical, social, and cultural factors, each operationalized through specific indicators. This approach revealed how natural capacity, infrastructure adequacy, activity diversity, and cultural–aesthetic qualities collectively shape the recreational value of the Bursa Atatürk Urban Forest. In this context, cultural factors serve as critical determinants of recreational outcomes, representing not only aesthetic experiences but also the symbolic, social, and identity-based connections that users actively establish with the space, thereby shaping engagement and overall experiential quality. Consequently, the findings identify the strengths and weaknesses of the site and provide a transferable model that can be applied in assessing the recreational performance of other urban forests.

Recreational planning in urban forests ensures the conservation and sustainability of nature-based areas and is crucial in enhancing urban quality of life and public health. Therefore, the recreational potential of urban forests must be assessed multidimensionally, considering ecological, physical, social, and cultural factors. A holistic and strategic evaluation model should be developed by integrating qualitative data with quantitative methods through multi-criteria decision-making (MCDM) techniques.

In this study, the term cultural parameters refers to human-influenced and anthropogenic features of the urban forest environment, including land use patterns, recreational facilities, accessibility features, and visual design elements shaped through planning and management interventions. These are distinguished from natural parameters, encompassing biophysical characteristics such as vegetation, topography, and hydrology. Visual aesthetics are classified as cultural because they primarily result from intentional design and maintenance choices rather than solely from natural processes.

In this context, the study conducted in Bursa Atatürk Urban Forest aimed to minimize the influence of subjective judgments and obtain more objective and measurable results through a weighted SWOT analysis. The performance score was calculated as 113.5, indicating a moderate level and falling short of the ideal score of 300. Within this model, cultural parameters such as land use and recreational facilities received high scores, while elements like disabled access and the lack of bicycle paths were rated poorly. This suggests that users’ cultural connections with the space are as significant as their opportunities for physical use. Factors such as the area’s historical continuity, its position within collective memory, visual symbols, and shared activity spaces play a pivotal role in shaping the perceived significance of cultural landscape values.

Furthermore, the weighted scores of natural and cultural data were compared using an independent samples *t*-test, revealing that the mean scores of cultural parameters were significantly higher than those of natural parameters (*p* ≤ 0.001). This indicates that users tend to evaluate the site primarily through the lens of human-made and socially functional features; however, these results are correlational and should not be interpreted as evidence of a causal relationship between cultural elements and higher performance. Underlying these preferences are cultural dynamics, including the integration of the urban forest into everyday life, its entrenchment in social memory, and the reinforcement of users’ place attachment and sense of belonging toward the space. Similarly, one-way ANOVA showed the highest scores were in recreational amenities, visual qualities, and other environmental factors. Although natural components (e.g., vegetation, drainage, elevation) were statistically significant, functional and aesthetic elements dominated user preferences. Disabled accessibility was the lowest-scoring criterion (0.75), indicating a critical deficiency in social equity and inclusivity. This situation suggests developing the area through social justice and inclusivity principles. Additionally, it should be considered that regulations ensuring equal access and participation for all user groups will contribute to the improvement of the overall public health status of the community. Moreover, given that cultural participation relies on physical infrastructure, deficiencies in accessibility pose limitations on the formation of cultural connections with the space. In addition, it should be considered that arrangements ensuring equal access and participation for all user groups will improve overall public health.

Additionally, a correlation analysis comparing SWOT parameters and weighted performance scores revealed a positive relationship between total scores and strengths and opportunities, whereas a negative correlation was observed with weaknesses. This indicates that improving weak aspects of the area would significantly enhance overall performance. Strengths and opportunities positively contribute to performance, while weaknesses suppress strengths and reduce the score.

In the Atatürk Urban Forest, cultural landscape elements play a more decisive role than natural landscape features. The proper and effective use of the area directly influences the level of benefit that users can derive from it. Therefore, preserving the strengths and improving the weaknesses related to cultural landscape elements is essential. In this context, addressing accessibility deficiencies—particularly by redesigning the area in line with universal design principles to accommodate individuals with disabilities—developing bicycle paths and alternative transportation infrastructure, enhancing visual and recreational amenities, and implementing activities and planning strategies that maintain the site’s attractiveness throughout the year by considering seasonal variations in use are of critical importance.

In conclusion, multi-factor evaluations and integrated methods for assessing recreational performance provide a scientific foundation for future strategic planning in urban green spaces. More effective and sustainable planning decisions can be achieved by analyzing recreational land uses through comprehensive models. Nonetheless, the distinction between natural and cultural parameters, as defined in this study, should be considered when interpreting these findings, and caution should be exercised to avoid inferring causation from observed correlations. In the operationalization of natural factors, defining indicators such as topographic structure, climatic characteristics, hydrological processes, vegetation cover, and soil components within a measurable framework, and in the operationalization of cultural factors, systematically applying criteria such as accessibility, land use categories, recreational infrastructure, safety conditions, and visual quality, strengthens the conceptual consistency of the model. Within this framework, rendering cultural landscape elements measurable through indicators such as visual quality, usage patterns, social activities, and symbolic values allows the cultural dimension to be assessed interpretatively and analytically. By defining natural and cultural landscape values through such clear and measurable indicators, the study presents a model that is transferable, adaptable, and conceptually consistent, applicable not only to Bursa Atatürk Urban Forest but also to other urban forests. The consideration of cultural factors in the recreational planning of urban forests emerges as a key element that deepens the user–space relationship, enhances social cohesion, and fosters a sense of belonging. In particular, the preservation of symbolic sites embedded in urban memory, the development of cultural infrastructures that accommodate the spatial experiences of diverse social groups, and the promotion of participatory design processes that reinforce social attachment are crucial for the sustainability of cultural landscape values. Such practices increase both the flexibility of the method and its potential for widespread adoption in the strategic planning of urban green spaces.

## Figures and Tables

**Table 1:** Evaluation of the recreational performance.

Natural Landscape Values—Geological and Geomorphological Structure, Topographic Structure, Natural Vegetation and Wildlife, Water Resources, Climatic Characteristics, Soil Structure				Current Status of The Area (Suitability Score)	Swot Analysis Based on Field Data Scoring				Average Swot Coefficient	Weighted Score
					Strengths (+) 4	Weaknesses (−) 2	Opportunities 3	Threats (−) 1		
Ecological Factors										
Factors (Factors refined through natural landscape values analyses)	Sub-Factors		Suitability Value (1–5)							
Slope	0–2 %		Highly Suitable—5	2	0	2	0	0	0.5	1
	2–6%		Suitable—4							
	6–2%		Moderate—3							
	12–20%		Low—2							
	20–30%		Very Low—1							
	Over 30%		Very Low—1							
Exposition	S-SE-SW-P		Highly Suitable—5	5	4	0	0	0	1	5
	E-W		Suitable—4							
	NE-NW		Moderate—3							
	N		Low—2							
Elevation Groups	0–250 M		Highly Suitable—5	5	4	0	0	0	1	5
	250–500 M		Suitable—4							
	500–750 M		Moderate—3							
	750–1000 M		Low—2							
	1000–1250 M		Very Low—1							
Erosion	None–Mild		Highly Suitable—5	5	0	2	0	0	0.5	2.5
	Moderate		Moderate—3							
	Severe		Very Low—1							
Drainage	No Drainage Issue		Highly Suitable—5	5	4	0	0	0	1	5
	Drainage Issue		Very Low—1							
Land Use Capability Class	Class VIII		Highly Suitable—5	3	0	2	0	0	0.5	1.5
	Class VII		Suitable—4							
	Class VI		Moderate—3							
	Class V		Low—2							
	Class IV		Very Low—1							
Climate	Temperature	15–25^0^C (Moderate)	Highly Suitable—5	3	0	2	0	1	0.75	2.25
		25–36 °C (hot) 4–15 °C (cold)	Moderate—3							
		< 4 °C (very cold) ˃36 °C (very hot)	Very Low—1							
	Rains	500–1250 MM	Highly Suitable—5	5	4	0	3	0	1.75	8.75
		250–500 MM 1250–1500 MM	Moderate—3							
		0–250 MM ˃1500	Very Low—1							
	Sunlight Average	8–10	Highly Suitable—5	4	4	0	0	0	1	4
		5–7	Suitable—4							
		2–4	Moderate—3							
		˃2	Low—2							
	Wind	˃1 m/s	Low—2	1		2			0.5	0.5
		1–3 m/s	Very Low—1							
Hyrology	Coastal Area		Highly Suitable—5	3	0	0	3	0	0.75	2.25
	Lake Shore		Suitable—4							
	Riverbank		Moderate—3							
Vegetation	Forest Areas		Highly Suitable—5	5	4	0	3	0	1.75	8.75
	Forest Openings and Open Areas		Very Low—1							
Geographical Location Area Size	>10 ha		Highly Suitable—5	5	4	0	0	0	1	5
	5–10 HA		Suitable—4							
	1–5 HA		Moderate—3							
	0.5–1 HA		Low—2							
**Cultural Landscape Values—Accessibility, Current Land Use,** **Other Environmental Characteristics, Boundaries- Security** **and Recreational Facilities**										
Factors (Factors refined through cultural landscape values analyses)	Sub-Factors		Suitability Value (1–5)							
**Physical Factors**										
Current Land Use	Forest Area		Highly Suitable—5	5	4	0	3	0	1.75	8.75
	Heathland		Suitable—4							
	Pasture-Grassland		Moderate—3							
	Garden		Low—2							
	Agricultural Land		Very Low—1							
Accessibility										
Proximity to Settlement	0–1 km		Highly Suitable—5	5	4	0	3	0	1.75	8.75
	1–2 km		Suitable—4							
	2–3 km		Moderate—3							
	Over 3 km		Low—2							
Road Type	Main Road		Highly Suitable—5	5	4	0	3	0	1.75	8.75
	Stabilized or Dirt Road		Moderate—3							
	Forest Road		Very Low—1							
Pedestrian Paths	Available; appropriate surface material, adequate width		Highly Suitable—5	1	0	2	0	1	0.75	0.75
	Available; inadequate width, inappropriate surface material		Moderate—3							
	Not Available		Very Low—1							
Bicycle Paths	Available; appropriate surface material, adequate width		Highly Suitable—5	1	0	2	3	0	1.25	1.25
	Available; inadequate width, inappropriate surface material		Moderate—3							
	Not Available		Very Low—1							
Parking Availability	Available		Highly Suitable—5	3	4	0	0	1	1.25	3.75
	The number of parking spaces is insufficient relative to user capacity.		Moderate—3							
	Not Available		Very Low—1							
Accessibility and usage opportunities for people with disabilities	Available		Highly Suitable—5	1	0	2	0	1	0.75	0.75
	Not Available		Very Low—1							
**Cultural Factors**										
Visual Values	**Scenic viewpoints** (urban landscape, forest landscape, etc.)		Highly Suitable—5	5	4	0	3	0	1.75	8.75
	The number of phytological formations, natural monuments, etc.		Highly Suitable—5							
Other Environmental Factors	Cleanliness of air, cleanliness of water, quiet and peaceful environment, and well-maintained conditions		Highly Suitable—5	3	4	0	0	1	1.75	8.75
	Regular cleaning and maintenance at specified intervals		Moderate—3							
	Presence of air, water, and noise pollution; lack of maintenance		Very Low—1							
**Social Factors**										
Security	Boundary elements define controlled entry and exit, the area’s boundaries.		Highly Suitable—5	4	0	2	0	0	0.50	2
	Only controlled entry and exit		Suitable—4							
	Undefined or unclear boundaries		Very Low—1							
Recreational Facilities	**Presence of Facilities and Amenities:** Food and beverage areas, picnic areas, viewing terraces, children’s playgrounds, and related equipment Seating and resting areas Food and beverage facilities and equipment Children’s playgrounds and play equipment Water features (fountains) Lighting fixtures Trash bins Shelter structures Signage and information boards Boundary elements Restrooms and infrastructure facilities Sales units		Highly Suitable—5	5	4	0	3	0	1.75	8.75
	Accommodation Areas (Permanent Overnight Stay/Camping/Caravan Sites)		Moderate—3							
	Insufficiency of Facilities for User Capacity		Low—2							
	Lack of Facilities		Very Low—1							
			TOTAL SCORE							113.5

**Table 2:** Results of the correlation analysis between SWOT parameters and score values (* *p* < 0.05 and ** *p* < 0.01).

	Score	Strengths	Weaknesses	Opportunities	Threats
Score	1				
Strenghts	0.824 **	1			
Weakness	−0.743 **	−0.871 **	1		
Opportunities	0.637 **	0.359	−0.384	1	
Threats	−0.043	0.037	0.028	−0.283	1

## Data Availability

All data generated or analyzed during this study are included in this manuscript.
